# Bifunctional Chloroplastic DJ-1B from *Arabidopsis thaliana* is an Oxidation-Robust Holdase and a Glyoxalase Sensitive to H_2_O_2_

**DOI:** 10.3390/antiox8010008

**Published:** 2019-01-01

**Authors:** Aleksandra Lewandowska, Trung Nghia Vo, Thuy-Dung Ho Nguyen, Khadija Wahni, Didier Vertommen, Frank Van Breusegem, David Young, Joris Messens

**Affiliations:** 1 VIB-VUB Center for Structural Biology, VIB, 1050 Brussels, Belgium; allew@psb.ugent.be (A.L.); Trung.Nghia.Vo@vub.be (T.N.V.); Ho.Thuy.Dung.Nguyen@vub.be (T.-D.H.N.); khadija.wahni@vib-vub.be (K.W.); 2 Brussels Center for Redox Biology, 1050 Brussels, Belgium; 3 Structural Biology Brussels, Vrije Universiteit Brussel, 1050 Brussels, Belgium; 4 Department of Plant Biotechnology and Bioinformatics, Ghent University, 9052 Ghent, Belgium; 5 VIB Center for Plant Systems Biology, VIB, 9052 Ghent, Belgium; 6 de Duve Institute, Université Catholique de Louvain, 1200 Brussels, Belgium; didier.vertommen@uclouvain.be

**Keywords:** chaperone, glyoxalase, holdase, redox

## Abstract

Members of the DJ-1 protein family are multifunctional enzymes whose loss increases the susceptibility of the cell to oxidative stress. However, little is known about the function of the plant DJ-1 homologs. Therefore, we analyzed the effect of oxidation on the structure and function of chloroplastic AtDJ-1B and studied the phenotype of T-DNA lines lacking the protein. In vitro oxidation of AtDJ-1B with H_2_O_2_ lowers its glyoxalase activity, but has no effect on its holdase chaperone function. Remarkably, upon oxidation, the thermostability of AtDJ-1B increases with no significant alteration of the overall secondary structure. Moreover, we found that *AtDJ-1B* transcript levels are invariable, and loss of AtDJ-1B does not affect plant viability, growth and stress response. All in all, two discrete functions of AtDJ-1B respond differently to H_2_O_2_, and AtDJ-1B is not essential for plant development under stress.

## 1. Introduction

α-dicarbonyls, such as glyoxal (GO) and methylglyoxal (MG), are toxic compounds produced during glycolysis, metal-catalyzed glucose auto-oxidation, and lipid peroxidation. When they react with proteins, they form advanced glycation end-products (AGEs), which have been implicated in the progression of diseases such as diabetes, atherosclerosis, and neurological disorders [1,2,3]. Plants also accumulate sugar-derived MG as a byproduct of the Calvin cycle [4]. GO and MG are being removed by a glutathione (GSH)-dependent two-enzyme system, consisting of glyoxalase I (GLYI) and glyoxalase II (GLYII) [5]. A third glyoxalase, initially termed GLYIII [6], is a GSH-independent enzyme. This GLYIII protein is a member of the DJ-1/ThiJ/PfpI superfamily, and it is conserved across all kingdoms. In humans and plants, this enzyme is termed DJ-1, and it is known to exhibit a variety of cellular functions. Human DJ-1 protects rat neurons against oxidative stress [7], has chaperone [8], glyoxalase [9], and deglycase activity [10], and forms a scaffold for RNA-associated proteins [11]. DJ-1 has a conserved cysteine (Cys106 in human DJ-1), which is essential for its glyoxalase activity [12], and is easily oxidized to sulfinic and sulfonic acid by H_2_O_2_ [13,14]. 

Humans have only one isoform of homodimeric DJ-1, while plants have six isoforms encoded by tandem repeats and resulting in DJ-1 pseudodimers with varying subcellular localization [15]. In plants, the role of these DJ-1 isoforms in protecting against oxidative stress is poorly understood. Among Arabidopsis DJ-1 proteins, the experimental evidence is limited to cytosolic AtDJ-1A and chloroplastic AtDJ1-C. AtDJ-1A protects the plant from high light by acting as an antioxidant enzyme and a copper chaperone for superoxide dismutase (SOD) [16]. However, the SOD chaperone activity of DJ-1 proteins has been disputed [17]. Another DJ-1 homolog, AtDJ-1C, was found to be essential for plant viability and chloroplast development [18]. Previously, we identified AtDJ-1A to be sulfenylated in vivo [19], and more recently we also identified AtDJ-1B as a redox-sensitive sulfenylated protein in the choroplasts [20].

In this study, we focus on AtDJ-1B, and evaluate the effect of H_2_O_2_ on the activity of this enzyme. We found that oxidation increases the thermal stability of AtDJ-1B, but inactivates its glyoxalase activity. We further demonstrated that AtDJ-1B is a holdase and that oxidation of AtDJ-1B has no effect on its chaperone activity. Finally, the analysis of two independent SALK T-DNA lines showed for the first time that the loss of AtDJ-1B does not affect the viability, growth, or stress response of Arabidopsis plants.

## 2. Materials and Methods 

A summarized table of buffers referred to in this section can be found in Supplementary Information. 

### 2.1. Cloning and Purification of Recombinant AtDJ-1B

An N-terminal Tobacco Etch Virus (TEV)-cleavage site was introduced into a codon-optimized Arabidopsis AtDJ-1B open reading frame by polymerase chain reaction (PCR), before subcloning into a pDEST15 expression vector with an N-terminal glutathione-S-transferase (GST)-tag (Gateway technology, Thermo Fisher Scientific, Waltham, MA, USA)). This expression vector was transformed into SHuffle^®^ T7 competent *E. coli* (New England Biolabs, Ipswitch, MA, USA) and plated on agar to obtain a single colony, which was grown overnight at 30 °C in Luria-Bertani Broth (LB) supplemented with 100 μg/mL ampicillin. 1 L Terrific Broth (TB, with 100 μg/mL ampicillin) cultures were inoculated with this overnight pre-culture and grown at 30 °C until they reached the exponential growth phase, then cooled to 20 °C, supplemented with 0.4 mM isopropyl-β-d-1-thiogalactopyranoside and further grown overnight at 20 °C.

Cells were pelleted and resuspended in Lysis Buffer, lysed in a cell cracker at 20 kilopounds per square inch, and centrifuged at 40,000 × *g* for 30 min, 4 °C. The supernatant was passed through a 0.45-μm filter and loaded onto Glutathione (GSH) Sepharose High Performance resin (GE Healthcare Europe, Diegem, Belgium) equilibrated with Binding Buffer A. After 1 h of incubation, unbound material was removed by washing the resin with 10 column volumes Binding Buffer, and the GST-tag was cleaved by incubating the resin with an 1 mg of TEV protease per estimated 10 mg of total bound protein overnight at 4 °C. Non-bound material (containing cleaved AtDJ-1B) was collected and incubated for 1 h at 4 °C with Ni^2+^-Sepharose 6 Fast Flow resin (GE Healthcare Europe, Diegem, Belgium) equilibrated with Binding Buffer B, for the purpose of capturing the His-tagged TEV protease. Non-binding protein was collected and concentrated by Vivaspin 20 (Sartorius, Göttingen, Germany) centrifugal filtration and injected onto a size-exclusion (SE) Superdex75 16/60 column (GE Healthcare Europe, Diegem, Belgium) equilibrated with SE Buffer. Protein eluates containing AtDJ-1B were collected and concentrated by centrifugal filtration, and protein concentration determined by Bradford assay [21]. Protein was then flash-frozen in liquid nitrogen and stored at −80 °C. 

### 2.2. Circular Dichroism (CD) 

Purified AtDJ-1B was buffer-exchanged into 50 mM 4-(2-hydroxyethyl)-1-piperazineethanesulfonic acid (HEPES) pH 7.3, 500 mM NaCl using Bio-Spin^®^ 6 columns (Bio-Rad Laboratories N.V., Temse, Belgium), the protein concentration measured by Bradford assay, and treated with either 5 mM tris(2-carboxyethyl)phosphine (TCEP), or with several H_2_O_2_ molar ratios (2:1, 10:1; 100:1). Treatments with TCEP or H_2_O_2_ were carried out at 25 °C for 1 h, after which samples were buffer-exchanged into degassed, filtered 20 mM sodium phosphate pH 7.4, 100 mM KF on Bio-Spin^®^ 6 (Bio-Rad Laboratories N.V., Temse, Belgium). These samples were centrifuged for 10 min at 20,000 × *g*, and the protein concentration was determined with the Bradford assay. Samples were vacuum-degassed for 10 min and CD spectra were collected at 20 °C using a J-715 spectropolarimeter (JASCO Inc., Easton, MD, USA) with a 0.1 mm cuvette from 250–185 nm using 1.0 nm sampling and averaged over 6 technical replicates. The spectrum of the buffer alone was subtracted from the resulting data, and the data converted to mean residue ellipticity. Spectra were deconvoluted by CONTIN ridge-regression analysis [22] within the DICHROWEB server [23,24], using a reference data set optimized for 190–240 nm.

### 2.3. Thermal Unfolding

The thermal stability of AtDJ-1B was assessed by monitoring the change in the absorbance of circularly polarized light at 222 nm as a function of temperature. Immediately following spectra acquisition by CD as described above, the same samples were subjected to a temperature gradient from 15–85 °C at a ramp of 1 °C min^−1^, and the absorption at 222 nm measured at 0.2 °C intervals. The linear slopes of the initial and final baselines of the sigmoidal unfolding curves were fitted with equation 1. For equation 1, *A* is ellipticity in mdeg (θ), *m* and *k* are the pre-unfolding intercept and slope respectively, *T* is temperature, *n* and *l* are the post-unfolding intercept and slope respectively.

(1) (A−(m+k∗T))/((n+l∗T)−(m+k∗T))

The resulting data of [U]/[F] was then plotted against T (Kelvin), and fitted with a Boltzmann Sigmoidal equation (Equation 2) in GraphPad Prism (version 7.0), where *y* is the data for [U]/[F], *slope* is the steepness of the curve, *x* is temperature in Kelvin, and *Tm* is the parameter for melting temperature (T_M_).

(2) $$y=y\min+\left(y\max-y\min\right)/\left(1+\exp\left(\frac{Tm-x}{\mathrm{slope}}\right)\right)$$

### 2.4. Glyoxalase Assay

The glyoxalase activity of AtDJ-1B was estimated by monitoring the degradation of its substrate, glyoxal, in function of time. The amount of glyoxal in the sample was measured by derivatizing it with 1,2-diaminobenzene, as described for methylglyoxal [25].

A reaction mix containing 7.62 mM glyoxal and 571 nM AtDJ-1B in Assay Buffer A was incubated for 2 h at 30 °C on a thermoblock with mixing. Every 20 min an aliquot of the reaction mix was taken, 2-fold diluted in Assay Buffer A, and 1,2-diaminobenzene (Sigma-Aldrich, Overijse, Belgium) and HClO_4_ were added to give a final concentration of 186 nM AtDJ-1B, 575 µM 1,2-diaminobenzene (Sigma-Aldrich, Overijse, Belgium) and 0.5 M HClO_4_. After 5 min mixing, the absorbance of the derivatized sample at 340 nm was measured. For each time point measurements from control standard solutions of 0, 2, 4, 6, 8, 10 mM of glyoxal derivatized in the same manner as the samples containing AtDJ-1B were taken for generation of a standard curve. The assays were performed for three experimental replicates with averaging of two technical replicates.

Purified AtDJ-1B was either reduced with 5 mM TCEP or oxidized by treatment with varying molar excess (2-fold, 4-fold, 6-fold, 8-fold, 10-fold, or 100-fold) of H_2_O_2_, or with 5 mM diamide (Sigma-Aldrich, Overijse, Belgium). After 1 h of incubation at 25 °C the oxidizing or reducing agents were removed by buffer exchange to Assay Buffer A using Bio-Spin^®^ columns (Bio-Rad Laboratories N.V., Temse, Belgium).

### 2.5. Chaperone Assay 

AtDJ-1B protein sample was buffer exchanged to Assay Buffer B and either reduced with 5 mM TCEP or oxidized by treatment with either 2-fold or 10-fold molar excess H_2_O_2_ to AtDJ-1B of 2:1 and 10:1 for 1 h at 25 °C. The samples were again buffer-exchanged into Assay Buffer B.

The thermal unfolding of citrate synthase (CS; from porcine heart; Sigma-Aldrich, Overijse, Belgium) was induced at 44 °C in Assay Buffer B with 0.24 μM citrate synthase (20 μg mL^−1^) and either 3-fold molar excess of Hsp90 (Sigma-Aldrich, Overijse, Belgium) as positive control, 5-fold molar excess of lysozyme (from chicken egg white, (Sigma-Aldrich, Overijse, Belgium)) as negative control, or 5-fold or 20-fold molar excess of AtDJ-1B protein. Aliquots (17 μL) were taken every 20 min and added to a cuvette containing the CS Assay Buffer to a final volume of 500 μL. Citrate synthase (CS) activity was followed by monitoring the rate of decrease of acetyl-CoA at 233 nm.

### 2.6. Oxidation of AtDJ-1B for Mass Spectrometric Analysis

AtDJ-1B was reduced with 50 mM TCEP for 30 min at room temperature and excess of TCEP was removed by Bio-Spin^®^ (Bio-Rad Laboratories N.V., Temse, Belgium) to a buffer containing 50 mM HEPES pH 7.3, 0.5 M NaCl. 73 µg of protein (25 µM) was mixed with 125-fold molar excess of dimedone prior to the addition of 10-fold molar excess of H_2_O_2_, and incubated at 37 °C for 1 h. H_2_O_2_ and dimedone were removed by Bio-Spin™ and the protein concentration determined. Iodoacetamide (IAM) was added at 400-fold molar excess (i.e., final concentration 8 mM) and the sample incubated for 1 h at room temperature in the dark.

### 2.7. Determination of Cysteine Oxidation States by Mass Spectrometry

For the identification of modified residues in AtDJ-1B, 10 µg of desalted proteins were denatured by methanol/chloroform precipitation and digested O/N with trypsin or chymotrypsin at 30 °C in 50 mM NH_4_HCO_3_ (pH 8.0).

After dissolving in eluent C (0.1% (*v*/*v*) trifluoroacetic acid in 2% (*v*/*v*) acetonitrile (ACN)), peptides were directly loaded onto reversed-phase pre-column (Acclaim PepMap 100, Thermo Fisher Scientific, Waltham, MA, USA) and eluted in backflush mode. Peptides were separated using a reversed-phase analytical column (Acclaim PepMap RSLC, 0.075 × 250 mm, Thermo Scientific), equilibrated in eluent A (0.1% (*v*/*v*) hydrofluoric acid (FA) with 4% eluent B (0.1% (*v*/*v*) FA in 80% (*v*/*v*) ACN, with a linear gradient of 4%–27.5% eluent B (0.1% hydrofluoric acid in 98% acetonitrile) for 100 min, 27.5%–40% eluent B for 10 min, 40%–95% eluent B for 1 min and holding at 95% for the last 10 min at a constant flow rate of 300 nl/min on an EASY-nLC 1000 ultra performance liquid chromatography (UPLC) system (Thermo Fisher Scientific, Waltham, MA, USA). Orbitrap Fusion Lumos tribrid mass spectrometer (Thermo Fisher Scientific, Waltham, MA, USA) was used for the analysis of the resulting peptides, which were then subjected to NanoSpray Ionization (NSI) source followed by tandem mass spectrometry (MS/MS) in Fusion Lumos coupled online to the UPLC. Orbitrap at a resolution of 120,000 was used for intact peptide detection. Peptides were selected for MS/MS using HCD (higher energy collisional dissociation) setting as 30; Orbitrap at a resolution of 30,000 was used for ion fragment detection. The the top 20 precursor ions above a threshold ion count of 5.0^3^ in the MS survey scan with 20.0 s dynamic exclusion were subjected to a data-dependent procedure that alternated between one MS scan followed by 20 MS/MS scans. The electrospray voltage applied was 2.1 kV. MS1 and MS2 spectra were obtained with an AGC target of 4E5 ions and a maximum injection time of 50ms and an AGC target of 5E4 ions and a maximum injection time of 100 ms, respectively. The *m/z* scan range for MS scans was 350 to 1500. MS/MS data processing was performed using Sequest HT search engine within Proteome Discoverer 2.2 against a homemade protein database containing the recombinant AtDJ-1A and AtDJ-1B sequences. Trypsin or chymotrypsin was specified as cleavage enzyme allowing up to 2 missed cleavages, 5 modifications per peptide, and up to 7 charges. Mass error was set to 10 ppm for precursor ions and 0.2 Da for fragment ions. Oxidation on Met, sulfenic-dimedone, sulfinic or sulfonic on Cys were considered as variable modifications. False discovery rate (FDR) was assessed using a fixed value PSM validator and thresholds for protein, peptide and modification site were specified at 1%. Site of covalent modification were manually validated. The mixed disulfides were identified by the use of the DBond software (Hanyang University, South Korea) [26].

### 2.8. Gene Expression Levels

The transcript level changes upon various treatments were obtained from several RNA Sequencing (RNA-Seq) experimental datasets, including: 3-h high light stress on *cat2-2* plants [27], treatment of Col-0 plants with 50 µM Antimycin A [28], Restricted Gas and Continuous Light (RGCL) treatment [29] of *cat2-2* or Col-0 plants for 24 h [30], 24 h of methyl viologen treatment of Col-0 plants (He et al., submitted) and *Pseudomonas syringae* infection of Col-0 plants (Stael et al., personal communication). Data visualization was performed using Heatmapper [31], genes were hierarchically clustered using Euclidean distance with average linkage.

### 2.9. Plant Material

All mutants used in this study are SALK T-DNA insertion lines in Col-0 background: *dj1a* (SALK_049637), *dj1b-4* (SALK_046449), *dj1b-9* (SALK_093414). All lines were genotyped prior to further analysis and homozygous plants were selected for phenotyping and confirmation of gene expression levels by RT-qPCR. 

### 2.10. RT-qPCR

To confirm that T-DNA lines are true knockout lines, their RNA was extracted from three-week-old plants rosettes using TRIzol solubilization and extraction [32] followed by RNeasy Plant Mini Kit (Qiagen, Venlo, The Netherlands). First strand cDNA synthesis was performed using iScript cDNA Synthesis Kit (Bio-Rad Laboratories N.V., Temse, Belgium) using 1 μg of total RNA used as input material. The 5-fold diluted cDNA and gene-specific primers (Supplementary Table) were used for RT-qPCR performed by iCycler iQ (Bio-Rad Laboratories N.V., Temse, Belgium), with SYBR Green I Master Kit according to manufacturer’s instructions. Data was analyzed by qBASE+ (Biogazelle, Zwijnaarde, Belgium), using *ELONGATION FACTOR 1α (EF-1α)* and *POLYUBIQUITIN 5 (UBIQ5)* as reference genes. For each data point three biological and three technical replicates were used. 

### 2.11. Growth Conditions and Plant Stress Assays

To assess the effect of high light, plants were grown in soil for 21 days in a controlled chamber (100 μmol·m^−2^·s^−1^ light intensity,16 h/8 h light/dark, 21 °C, 50% relative humidity). Three-week-old plants were transferred o high light (600 μmol·m^−2^·s^−1^) for 72 h. The bright-light pictures of the plants, as well as measurements of photosystem II (PSII) maximum efficiency (Fv’/Fm’) using an Imaging- PAM-Series chlorophyll fluorescence system (HeinzWalz, Effeltrich, Germany) were taken every 24 h.

To assess mutants’ response to various stress conditions in vitro, plants were surface-sterilized by fumigation, vernalized for 3–4 days at 4 °C and grown under controlled conditions (16 h/8 h light/dark, 100 μmol·m^−2^·s^−1^ light intensity, 21 °C, 70% relative humidity) for 21 days. The medium was half-strength Murashige-Skoog medium (½ MS, 1% *w*/*v* sucrose, 0.8% *w*/*v* agar) containing one of the following additives: 25 mM mannitol, 50 mM NaCl, 25 nM methyl viologen or 1 μM 3-aminotriazole (3-AT). Plants were also grown on control plates, i.e., ½ MS with no additives. Plant rosette measurements were performed using ImageJ software [33] from 9 until 19 days after germination.

The Restricted Gas exchange and Continuous Light (RGCL) treatment was used to trigger photorespiratory stress in plants growing on ½ MS agar plates [34]. 21 days after vernalization, plates (either ½ MS or ½ MS + 3-AT) were sealed with multiple layers of Parafilm^®^ M (Bemis Company Inc., Oshkosh, WI, USA) in order to restrict gas exchange and transferred to continuous light (100 μmol·m^−2^·s^−1^ light intensity, 21 °C, 70% relative humidity) for 10 days. The bright-light pictures of the plants, as well as measurements of photosystem II (PSII) maximum efficiency (Fv’/Fm’) using an Imaging- PAM-Series chlorophyll fluorescence system (HeinzWalz, Effeltrich, Germany) were taken every 2–3 days.

## 3. Results

### 3.1. AtDJ-1B Contains Multiple Oxidant-Sensitive Cysteines 

**Figure 1:**
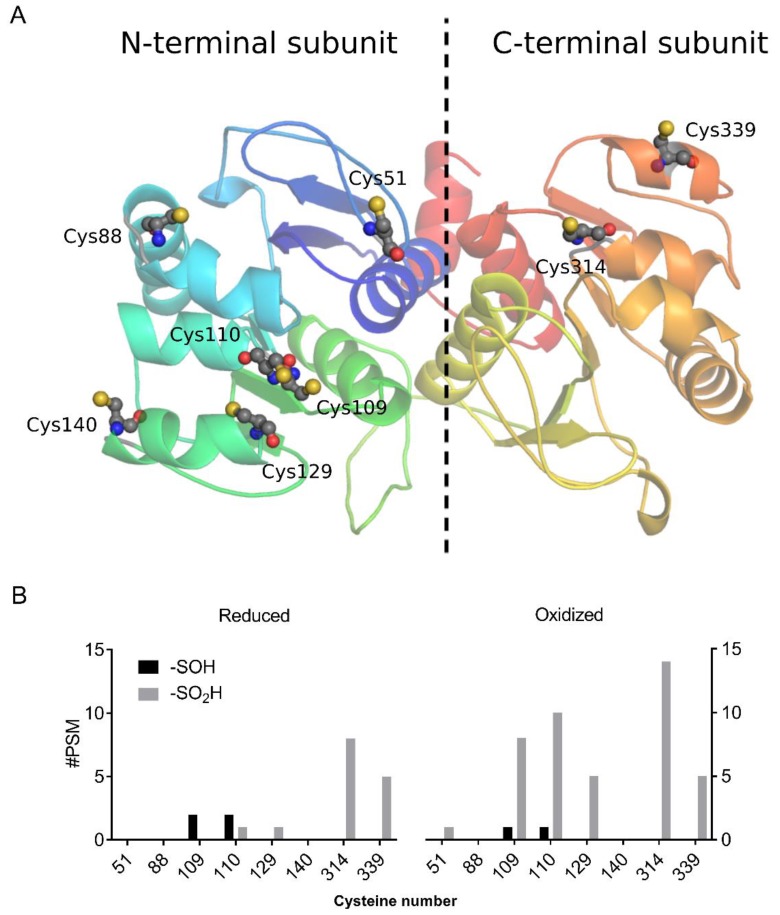
AtDJ-1B cysteines are differentially oxidized upon H_2_O_2_ treatment. (**A**) Homology model of AtDJ-1B with annotated cysteines in ball-and-stick representation. A dotted line approximately marks the 2-fold pseudosymmetry axis arising from the pseudo-dimeric AtDJ-1B monomer. Model generated using the I-TASSER structural prediction server, wherein the crystal structure of AtDJ-1D (PDB ID, 3UK7) was applied as a threading template. (**B**) Number of peptides (#PSM) detected by mass spectrometry that contained sulfenylated (-SOH) and sulfinylated (-SO_2_H) cysteines, either in a sample reduced by 5 mM TCEP or oxidized by 10-fold molar excess of H_2_O_2_ for 1 h.

The conserved catalytic cysteine of human DJ-1 is recognized as being highly prone to oxidation, with the most commonly observed oxidation state of this cysteine being sulfinic acid [35]. Arabidopsis AtDJ-1B is sulfenylated in planta [20], but nothing was known about its sulfenylation sites. AtDJ-1B is a pseudodimer containing two conserved DJ-1/PfpI domains in which there are eight cysteines, six within the N-terminal subunit, and two in the C-terminal subunit (Figure 1A). Of these eight cysteines, two correspond to the conserved catalytic cysteines of homodimeric human DJ-1 (Cys109 and Cys314), and seven are predicted to be solvent-exposed based on a homology model of AtDJ-1B (Cys129 being the sole buried residue). Hence, we sought to characterize the extent of cysteine oxidation in recombinantly expressed AtDJ-1B after exposure to H_2_O_2_. Mass spectrometry (MS) analysis of AtDJ-1B treated for 1 h with a 10-molar excess of H_2_O_2_ showed, as expected, sulfinylation of the conserved active-site cysteines (Cys109 and Cys314) (Figure 1B). Sulfinylation also occurred at Cys110, and to a lesser extent at Cys129 and Cys339 (Figure 1B). 

### 3.2. Oxidized AtDJ-1B Becomes More Thermostable

**Figure 2:**
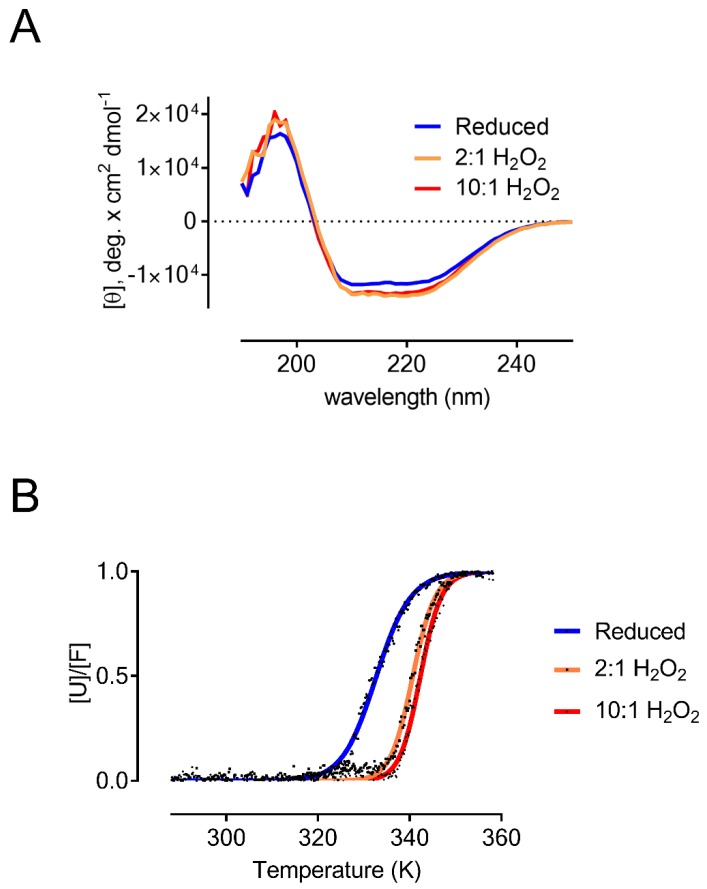
Oxidation does not influence the secondary structure of AtDJ-1B but increases its thermostability. (**A**) Comparative CD spectra of AtDJ-1B reduced with TCEP (blue trace), and DJ-1B oxidized by treatment with either a 2-fold (orange) or 10-fold (red) molar excess of H_2_O_2_. A slight spectral shift can be observed for the H_2_O_2_-treated protein relative to the reduced protein, though this spectral difference does not ultimately relate to any significant change in secondary structure. (**B**) Thermal unfolding of AtDJ-1B fitted to the Gibbs-Helmholtz equation. The change in ellipticity at 222 nm was followed by CD as a function of temperature for reduced AtDJ-1B (5 mM TCEP), and oxidized AtDJ-1B (either 2-fold, or 10-fold molar excess of H_2_O_2_). Data were converted to fraction of unfolded protein ([U]/[F]) and baseline-subtracted as described in Materials & Methods.

To assess the effect of the redox state of AtDJ-1B on its structural stability, we used circular dichroism (CD). We compared the relative secondary structure content of reduced and oxidized AtDJ-1B using increasing molar ratios of H_2_O_2_ (Figure 2A). The secondary structure of AtDJ-1B was not significantly altered upon oxidation, and both reduced and oxidized AtDJ-1B are characterized by 36–37% α-helical and 15–17% β-strand content, in agreement with previous findings, and comparable to what was observed for human DJ-1 [36]. 

To evaluate the effect of oxidation on the thermostability of AtDJ-1B, we determined the melting temperature (T_M_) of AtDJ-1B by following the change in ellipticity at 222 nm in function of temperature (Figure 2B). After fitting the data with a Boltzmann Sigmoidal equation, we obtained a T_M_ of 59.7 °C for reduced AtDJ-1B. For oxidized AtDJ-1B, the T_M_ increased to 67.6 °C and 69.3 °C following treatment with 2-fold and 10-fold molar excess of H_2_O_2_, respectively. This demonstrates that the oxidized form of AtDJ-1B is significantly more thermostable than the reduced form. 

### 3.3. Oxidation Inactivates the Glyoxalase Activity of AtDJ-1B

**Figure 3:**
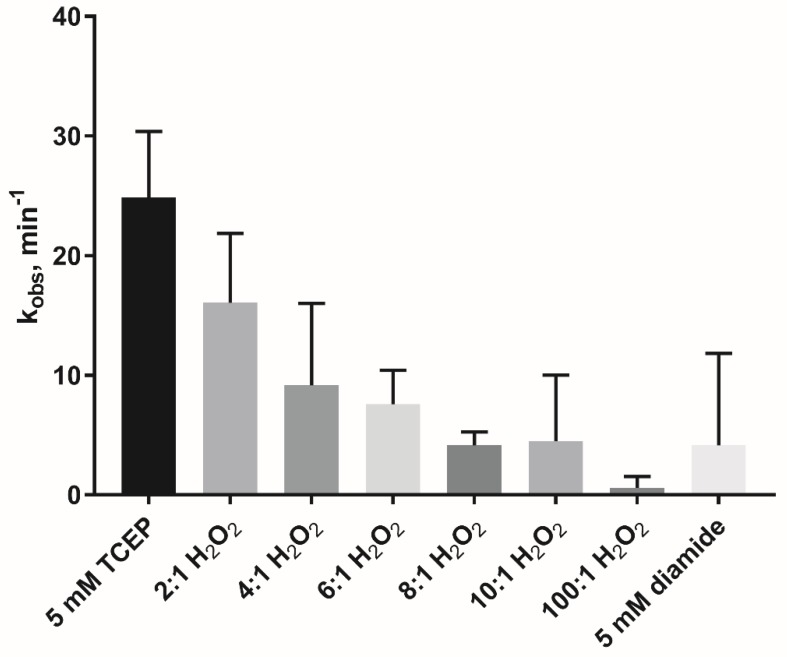
Glyoxalase activity of AtDJ-1B is lost after oxidation. Reduced and oxidized AtDJ-1B were prepared by pretreatment with either TCEP, varying molar ratios of H_2_O_2_: protein, or diamide. The reductant/oxidant was then removed prior to the activity assay. Displayed are observed rate constant (k_obs_) values averaged from experimental triplicates with ± standard deviation (SD) indicated.

One of the demonstrated functions of DJ-1 proteins is as a GSH-independent glyoxalase enzyme [9,37,38]. To measure the glyoxalase activity of AtDJ-1B, we monitored the consumption of glyoxal in function of time through derivatization with 1,2-diaminobenzene (Figure 3). Then, under near-steady-state conditions (8 mM glyoxal) the effect of increasing molar ratios of H_2_O_2_ on the glyoxalase activity of AtDJ-1B was evaluated. Pre-treatment of AtDJ-1B with a 2-fold molar excess H_2_O_2_ resulted in a decrease of the relative activity to ~64% of the activity of the reduced enzyme, and a further decrease to ~16% after treatment with 10-fold molar excess H_2_O_2_. This dose-dependent oxidative inactivation of AtDJ-1B was further confirmed by complete inactivation of glyoxalase activity following treatment with a 100-fold molar excess of H_2_O_2_. Oxidation of AtDJ-1B by treatment with 5 mM diamide (disulfide bond formation) also elicited a decrease to ~17% of full activity. 

### 3.4. Oxidation does not Affect the Chaperone Activity of AtDJ-1B

**Figure 4:**
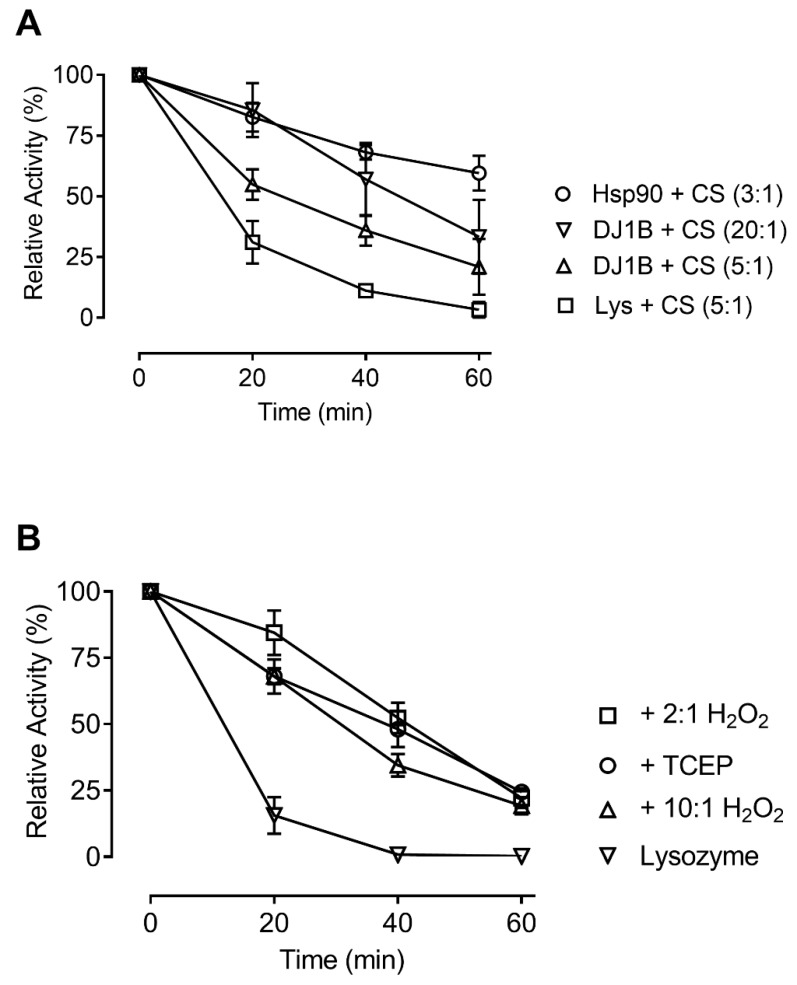
Suppression of thermal inactivation of citrate synthase (CS) by AtDJ-1B is independent of the oxidation state of AtDJ-1B. (**A**) At 44 °C, CS was incubated with 5-fold molar excess of lysozyme, 3-fold molar excess of Hsp90, 20- or 5-fold molar excess of DJ-1B at 44 °C, and activity measurements taken every 20 min for 1 h. (**B**) CS was incubated with 5-fold molar excess of lysozyme, or 20-fold molar excess of DJ-1B, either reduced with 5 mM TCEP, or oxidized with 10:1 or 2:1 molar excess of H_2_O_2_ at 44 °C, and activity measurements taken every 20 min for 1 h. Given is relative activity as a percentage of the full enzymatic activity of CS, just prior to heat-block incubation. Each data point represents average ± SD for triplicates.

Hsp31, a close homolog of AtDJ-1B in *Saccharomyces cerevisiae*, has previously been demonstrated to function as a holdase and confer stress resistance [39,40,41]. To explore whether AtDJ-1B is capable of similar functionality, we assessed the ability of AtDJ-1B to act as a holdase towards citrate synthase (CS). Here we found that AtDJ-1B protects CS against thermal inactivation at 44 °C, with 5-fold molar excess of AtDJ-1B preserving 35% of CS activity after 40 min heat treatment compared to only 10% preservation when using a 5-fold molar excess lysozyme as a negative control (Figure 4A). Although AtDJ-1B offered less protection than the positive control of Hsp90 (preserving 68% of activity at a 3-fold molar excess), the extent to which AtDJ-1B protected CS against inactivation was proportional to the relative concentration of AtDJ-1B, with 20-fold molar excess resulting in greater protection than 5-fold excess (Figure 4A). Considering a putative role of AtDJ-1B in the oxidative stress response, we evaluated the impact of oxidation on the ability of AtDJ-1B to act as a holdase. Remarkably, no significant difference was observed between the chaperone effectiveness of reduced (TCEP-treated) and oxidized (H_2_O_2_-treated) AtDJ-1B, with all samples showing a similar level of protection against thermal inactivation of CS (Figure 4B).

### 3.5. DJ-1B-Deficient Plants are Phenotypically Identical to Wildtype 

Having established the possible activities of DJ-1B and their redox regulation, we sought to determine the phenotype of Arabidopsis T-DNA *dj-1b* insertion lines. Two SALK T-DNA *dj1b* lines (further referred to as *dj1b-4* and *dj1b-9*, see Materials and Methods) and one SALK T-DNA *dj-1a* line were genotyped and confirmed to be homozygous. The *dj1b-4* and *dj1b-9* mutants contain the T-DNA insert in the 4th exon and the 4th intron, respectively, while in the *dj1a* line the insert is located in the promoter region (Supplementary Figure S1). The lack of gene transcripts was validated by reverse transcription quantitative PCR (RT-qPCR) (see Supplementary data).

**Figure 5:**
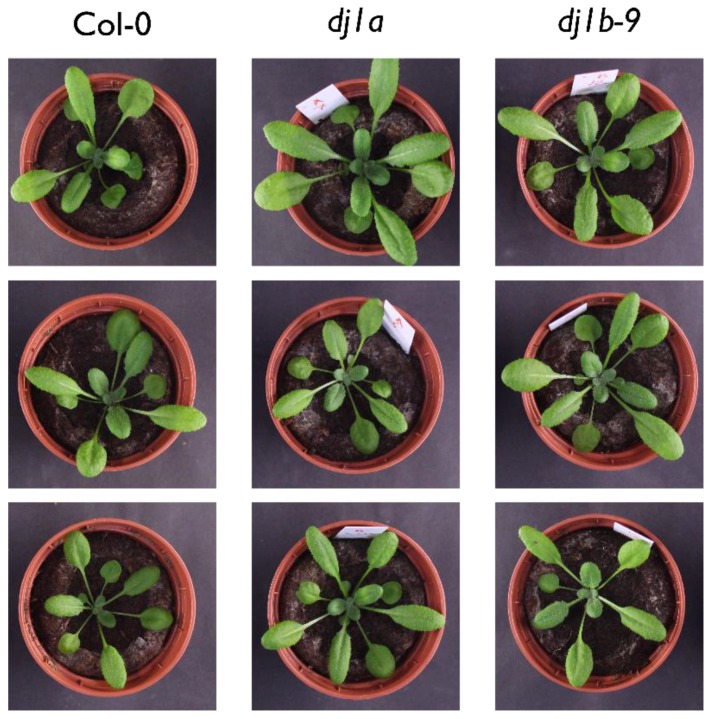
3-week old plants lacking AtDJ-1A or AtDJ-1B are phenotypically identical to wildtype Col-0 plants. Col-0, *dj1a* and *dj1b-9* plants were grown for 3 weeks in a long-day light regimen (16 h/8 h light/dark, 21 °C for 3 weeks. Representative bright-light images were taken 21 days after sowing.

**Figure 6:**
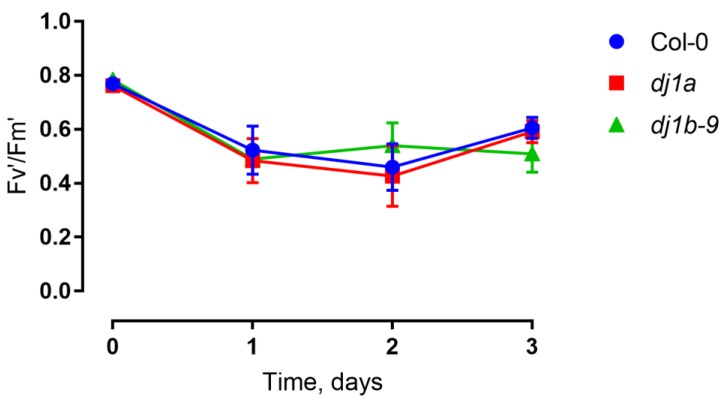
Photosystem II efficiency of mutants subjected to high light treatment was the same as that of wild type (WT). Three-week-old plants were exposed to high light intensities (600 μmol·m^−2^·s^−1^) for 72 h and the Fv’/Fm’ ratios were measured. Each data point represents average from biological triplicates ± SD.

When grown in soil in a growth chamber (16 h of light, 8 h of dark, 100 μmol·m^−2^·s^−1^ light intensity), the *dj1b* and *dj1a* mutants were phenotypically identical to wildtype Col-0 plants (Figure 5). Since *dj1a* mutants were previously reported to show increased susceptibility to high light stress [16], we sought to confirm these findings and checked whether plants lacking AtDJ-1B show the same phenotype. After 3 days of high light stress treatment, both wildtype and mutant plants had no leaf lesions and their photosystem II efficiency, inferred from Fv’/Fm’ ratios, remained unchanged (Figure 6).

**Figure 7:**
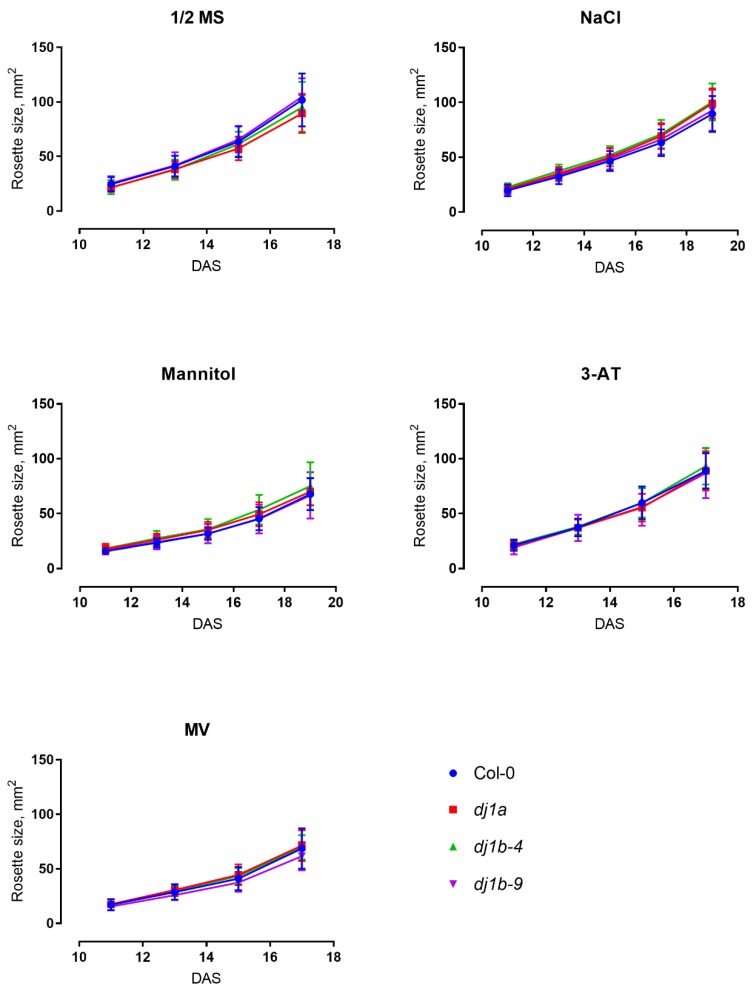
T-DNA insertion lines lacking AtDJ-1A or AtDJ-1B have no growth defects when subjected to various stresses. The plants were grown on ½ MS medium with stress-triggering additives: NaCl, mannitol, 3-amino-1,2,4-triazole (3-AT) or methyl viologen (MV) and their rosette sizes were analysed from bright-light images using ImageJ Software. Data points represent biological replicates ± SD.

**Figure 8:**
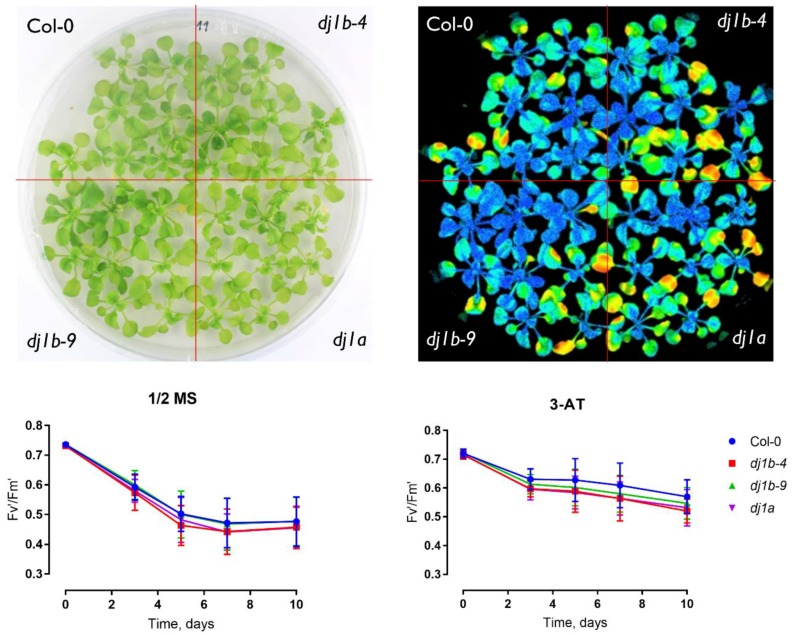
Plants deficient in AtDJ-1A orAt DJ-1B show no photorespiratory phenotype. (Upper panel) T-DNA *dj1a* and *dj1b* insertion lines do not differ from WT, as shown on a representative bright-light image (left) and color-coded Fv’/Fm’ image (right) after 7 days of Restricted Gas exchange and Continuous Light (RGCL) treatment. (Lower panel) Fv’/Fm’ decrease during the RGCL treatment. Data points represent averages from biological replicates ± SD.

To further characterize the phenotypes of *dj1a* and *dj1b* mutants, we grew them on ½ Murashige-Skoog (MS) agar medium for 3 weeks and assessed their rosette sizes when plants were subjected to different stress agents in the medium: NaCl, mannitol, methyl viologen, 3-amino-1,2,4-triazole. In all cases, mutants grown on control ½ MS medium or subjected to stress were characterized by the same rosette size as wildtype (Figure 7). Moreover, when plants were subjected to photorespiratory stress by a Restricted Gas exchange and Continuous Light (RGCL) treatment [34] for up to 10 days, the Fv’/Fm’ ratio of the mutants was the same as for wildtype (Figure 8). Taken together, these results indicate that the loss of DJ-1A or DJ-1B does not result in altered growth rates and photosynthetic capacities when exposed to the described abiotic stress conditions. 

### 3.6. Transcript Levels of DJ-1B are Stress-Independent

**Figure 9:**
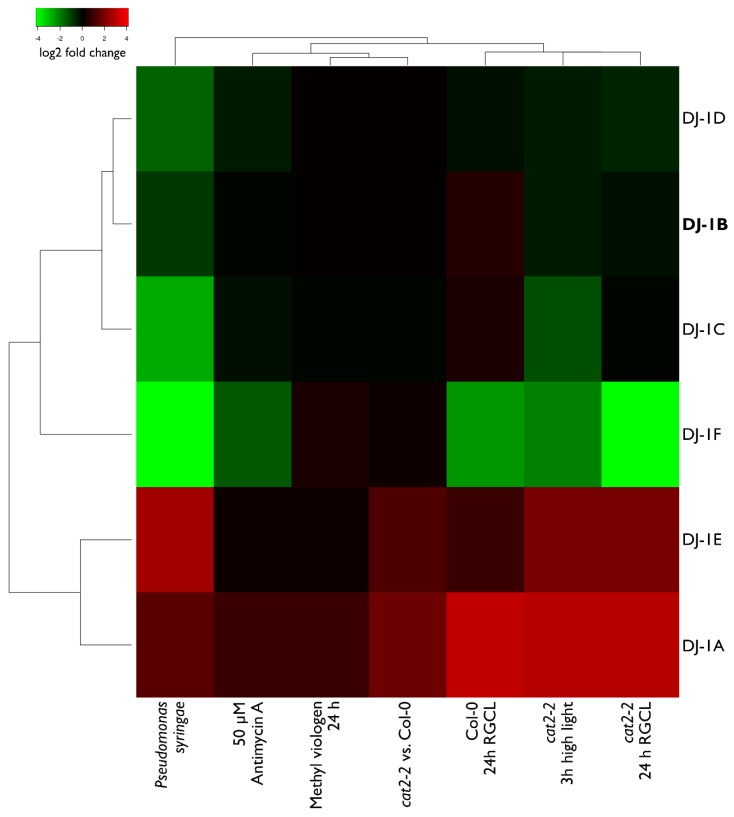
Arabidopsis DJ-1 gene expression levels respond differently after various stress treatments. Arabidopsis plants were either treated by a stress agent (pathogen *Pseudomonas syringage*, methyl viologen, high light, RGCL—Restricted Gas Continuous Light—treatment [29], or two lines (Col-0 and *cat2-2*) were compared to each other. The RNASeq experiments from which the data was obtained, as well as the detailed descriptions of the treatments are referred to in Materials and Methods. Colors represent log2 fold change.

Since various oxidative stress treatments trigger transcriptome-wide changes in Arabidopsis mRNA levels [42], we analyzed several RNA-Seq datasets, aiming to determine whether mRNA levels of all Arabidopsis DJ-1 homologs are also susceptible to such treatments (Figure 9). We concentrated on treatments triggering oxidative stress, such as RGCL [34], high light [27], methyl viologen treatment, and *Pseudomonas syringae* infection. In addition to wildtype Col-0, we also analyzed the transcriptome of *cat2-2* mutants, which are commonly used as stress-inducible systems to study oxidative stress in vivo [43]. 

The RNA-Seq dataset analysis confirmed the earlier finding that AtDJ-1A is upregulated under stress [16]. The same trend was observed for AtDJ-1E, whose mRNA levels were also upregulated in the majority of the stress conditions. Remarkably, for AtDJ-1F, which shares 76% protein sequence identity with AtDJ-1E, the mRNA levels are significantly decreased upon stress. The mRNA levels of the other three AtDJ-1 homologs are less susceptible to stress treatments; in particular AtDJ-1B transcription levels are not affected. This result is in line with previous studies of plant DJ-1 homologs showing different expression patterns and transcriptional responses to stress [44].

## 4. Discussion

We report reduced AtDJ-1B as a glyoxalase with specific activity of 600 nmol·min^−1^·mg protein^−1^ (Supplementary Table). For non-reduced AtDJ-1B, a specific activity of 310 nmol·min^−1^·mg protein^−1^ was reported [12]. The glyoxalase activity of AtDJ-1B is ~35 times lower than for AtDJ-1D when glyoxal is used as substrate (Supplementary Table) [12]. This lower glyoxalase activity could be due to the absence of a conserved histidine, which has been suggested to facilitate proton transfer and glyoxal stereospecificity [45]. In plants under biotic and abiotic stress, and after H_2_O_2_ treatment, the intracellular concentration of glyoxals has been shown to increase from 100 to 2000 µmol/g tissue [46,47]. When methylglyoxal is photoreduced by photosystem I during photosynthesis, it donates electrons to O_2_, producing superoxide (O_2_^•−^) [48]. Thus, by removing superoxide-generating glyoxals, enhanced glyoxalase activity contributes to cellular protection during oxidative stress. However, we clearly showed inactivation of the glyoxalase activity of AtDJ-1B by H_2_O_2_, the predominant oxidant under oxidative stress conditions (Figure 4). To our knowledge, this is the first report of a DJ-1 protein losing its glyoxalase activity upon oxidation. Moreover, as the GLYI/II enzyme system catalyzes glyoxal/methylglyoxal detoxification with far greater catalytic efficiency than even AtDJ-1D [49], we propose that in plants the majority of glyoxals is detoxified by GLYI/II enzymes, rather than by H_2_O_2_-sensitive DJ-1 proteins. 

AtDJ-1C has been reported with no glyoxalase activity [12], and its knockout results in non-viable plants [18]. Therefore, we sought to explore possible alternative functions for AtDJ-1B, which shares its chloroplastic localization with AtDJ-1C. We found that AtDJ-1B acts as an oxidation-robust holdase, though a molar excess of AtDJ-1B is needed to protect CS from thermal inactivation. Since porcine CS used for the holdase activity assay is not its natural client protein, it still remains unclear whether the holdase function of AtDJ-1B would be its real physiological function. Furthermore, it is known that dedicated chaperones suppress aggregation of client proteins at stoichiometric concentrations [50], whereas AtDJ-1B showed holdase activity at 5 to 20-fold molar excess. Similarly to human DJ-1 [8], AtDJ-1B could have specific client proteins in Arabidopsis that still have to be identified. 

Furthermore, human DJ-1 has been demonstrated to be a redox-dependent holdase, with the oxidized form of DJ-1 able to suppress α-synuclein aggregation significantly better than the reduced form, offering 20 to 60% more protection [8]. On the other hand, the yeast homolog Hsp31 is a redox-independent holdase and this chaperone activity is required for protecting yeast from cytotoxic stress [39]. We also found the holdase activity of AtDJ-1B to be redox-independent. Remarkably, we observed that the thermostability of AtDJ-1B increases upon oxidation, which indicates that the oxidized form of AtDJ-1B is more likely than reduced AtDJ-1B to retain its chaperone activity under sustained heat stress. Other DJ-1 family members have also been shown to be highly thermostable, with a T_M_ values ranging from 64 to 77 °C [36,51,52]. Here, we determined a comparatively lower T_M_ for AtDJ-1B of 59.7 °C, and observe an increase in T_M_ of approximately 8–10 °C for oxidized AtDJ-1B. Similar increase in thermostability has also been observed for human DJ-1 and drosophila DJ-1β, with respective T_M_ increases of 13.3 and 11.5 °C upon oxidation in the presence of a 7-fold molar excess of H_2_O_2_ [51]. 

Finally, it is worthy of mention that both human and prokaryotic DJ-1 homologs also have deglycase activity [53,54,55], and it was proposed to be one of the core physiological functional roles of DJ-1 proteins [56]. As glyoxalase function is required for deglycase activity, when glyoxalase activity is lost upon oxidation, the deglycase function will also be abolished. In both its role as a deglycase and as a holdase, it is reasonable to assume that AtDJ-1B has preferential target proteins. Further understanding of the in vivo role of AtDJ-1B will require the identification of its interaction partners in Arabidopsis. 

In contrast to AtDJ-1C, whose loss results in infertile plants with numerous developmental defects [18], AtDJ-1B loss does not influence plant viability. We also found that *dj1b* plants show wild-type resistance to stress, at least under the conditions tested. Moreover, while it was previously reported that AtDJ-1A deletion leads to increased susceptibility to high light [16], the phenotype of the *dj1a* SALK T-DNA line described here is identical to wildtype, which directly contradicts earlier findings [16].

The Arabidopsis DJ-1 family originates from one ancestor gene and diverged into six genes through both whole-genome and tandem duplications [57]. The same mechanism is responsible for higher duplication rates in plants in general, when compared to other eukaryotes [58,59]. After duplication, proteins evolve new functions; one of the copies might acquire a novel function, or if the ancestral protein is multifunctional, the duplicates divide the original function, for instance by differential expression patterns [59]. The analysis of RNASeq data presented in this paper shows that Arabidopsis DJ-1 proteins differ greatly in their expression patterns. These differences will be better understood after the analysis of their cis-regulatory elements in promoter sequences, like described for other plant species [44,60,61]. Moreover, since the mRNA levels are not representative for the protein levels, especially during dynamic phases such as cellular differentiation and stress response [62], it would be very informative to quantify all DJ-1 protein levels, both in stress and control conditions. Generating multiple mutant lines and biochemically characterizing all six Arabidopsis DJ-1 proteins will explain to what degree their functions overlap and whether the findings about the role of AtDJ-1B presented here are applicable to the other homologs as well. 

## 5. Conclusions

In summary, we showed for the first time that Arabidopsis DJ-1B is a bifunctional protein, having both glyoxalase and holdase activity. Importantly, these two functions are differently regulated by H_2_O_2_; while the glyoxalase activity is lost upon oxidation, the holdase activity is not affected by H_2_O_2_. We also, for the first time, report the *dj1b* plant phenotype and prove that AtDJ-1B is not necessary for viability, development, or stress resistance of Arabidopsis plants, which might be due to redundant functions of all DJ-1 homologs. To explain why Arabidopsis has retained six DJ-1 homologs, the physiological relevance of the bifunctionality of AtDJ-1B still need to be confirmed in vivo and compared to those of the other AtDJ-1 proteins.

## Supplementary Materials

The following are available online at http://www.mdpi.com/2076-3921/8/1/8/s1, Figure S1: *AtDJ-1A* and *AtDJ-1B* gene models, Figure S2: *AtDJ-1B* and *DJ-1A* transcript levels (left and right, respectively) in WT and KO T-DNA lines, Table S1: Component summary of buffering solutions used, Table S2: AtDJ-1B specific glyoxalase activities and corresponding observed reaction rates determined during glyoxalase assay, Table S3: Arabidopsis thaliana DJ-1 specific activities [12], Table S4: Primers used for the study, Table S5: Log2 fold change values of DJ-1 mRNA expression levels, as visualised on Figure 9.
