# Supplementary material for: Bifunctional Chloroplastic DJ-1B from Arabidopsis thaliana is an Oxidation-Robust Holdase and a Glyoxalase Sensitive to H2O2
Source: Antioxidants (Basel). 2019 Jan 1;8(1):8. doi: 10.3390/antiox8010008 (PMC6356872; doi:10.3390/antiox8010008)
Supplement: Supplementary file 1 [file antioxidants-08-00008-s001.pdf]

Table S1. Component summary of buffering solutions used.

Name	Chemical Ingredients
Lysis Buffer	50 mM 4-(2-hydroxyethyl)-1-piperazineethanesulfonic acid (HEPES)/NaOH, pH 7.3, 0.5 M NaCl, 2 mM dithiothreitol (DTT), 1 μg/mL leupeptine, 0.1 mg/mL 4-(2-aminoethyl) benzenesulfonyl fluoride hydrochloride (AEBSF), 50 μg/mL DNaseI, and 20 mM MgCl
Binding Buffer A	50 mM HEPES/NaOH, pH 7.3, 0.5 M NaCl, 5 mM DTT
Binding Buffer B	50 mM HEPES/NaOH, pH 7.3, 0.5 M NaCl, 30 mM imidazole, 1 mM DTT
SE Buffer	50 mM HEPES/NaOH, pH 7.3, 0.5 M NaCl, 1 mM tris(2-carboxyethyl)phosphine (TCEP)
Assay Buffer A	50 mM HEPES/NaOH, pH 7.3, 500 mM NaCl
Assay Buffer B	20 mM HEPES/NaOH, pH 7.3, 20 mM NaCl
CS Assay Buffer	40 mM HEPES/NaOH, pH 7.8 40 μM Acetyl-CoA (Sigma-Aldrich), oxaloacetic acid (Sigma-Aldrich), 20 mM KOH, 50 mM KCl and 10 mM $(NH_4)_2SO_4$

Table S2. AtDJ-1B specific glyoxalase activities and corresponding observed reaction rates determined during glyoxalase assay.

AtDJ-1B Treatment	Specific Activity (nmol·min^{-1}·mg protein^{-1})	k_{obs} (min^{-1})
**5 mM TCEP**	596 ± 115	24.9 ± 4.8
**2:1 H_2O_2**	385 ± 120	16.1 ± 5.0
**4:1 H_2O_2**	220 ± 134	9.2 ± 5.6
**6:1 H_2O_2**	182 ± 56	7.6 ± 2.3
**8:1 H_2O_2**	99 ± 22	4.1 ± 0.9
**10:1 H_2O_2**	108 ± 108	4.5 ± 4.5
**100:1 H_2O_2**	14 ± 19	0.6 ± 0.8
**5 mM diamide**	99 ± 151	4.1 ± 6.3

Table S3. Arabidopsis thaliana DJ-1 specific activities [12].

AtDJ-1 Isoform	Specific Activity (nmol·min^{-1}·mg protein^{-1})	
	Methylglyoxal	Glyoxal
**A**	15 ± 4	250 ± 12
**B**	13 ± 3	310 ± 5
**C**	–	–
**D**	8600 ± 200	11 000 ± 600
**E**	6.2 ± 1.7	–
**F**	5.5 ± 1.1	–

AtDJ-1B open reading frame sequence without N-terminal chloroplastic targeting sequence and start codon, optimized for *E. coli.*

(Gene ID At1g53280)

```
GCGACGATGTCGAGCAGTACGAAAAAGGTACTTATTCCCGTTGCCCATGGAACAGAGCCGTTTGA
AGCAGTCGTTATGATCGATGTATTGCGCCGTGGAGGTGCCGACGTAACAGTAGCGTCCGTTGAAA
ATCAAGTGGGCGTTGATGCATGCCACGGTATCAAAATGGTAGCCGACACGCTTCTGAGCGATATC
```

```
ACCGATAGCGTGTTCGACTTGATCATGTTACCCGGCGGGCTGCCTGGAGGTGAGACGCTGAAAAA
TTGCAAGCCGCTTGAAAAAATGGTTAAGAAACAAGACACTGACGGGCGCTTGAACGCAGCAATCT
GCTGTGCTCCGGCCTTAGCATTTGGCACTTGGGGTTTACTGGAAGGGAAGAAAGCAACGTGCTAT
CCTGTGTTCATGGAGAAGTTAGCCGCCTGTGCTACAGCTGTAGAATCTCGTGTCGAGATCGACGG
AAAAATTGTTACGAGTCGCGGACCCGGGACCACGATGGAATTCTCGGTGACACTTGTAGAGCAGT
TATTGGGTAAAGAGAAGGCGGTGGAAGTTTCAGGGCCCCTGGTTATGCGCCCGAACCCAGGAGAC
GAGTACACAATCACGGAGCTTAATCAAGTATCCTGGTCTTTCGAGGGGACACCTCAAATCCTTGT
CCCCATTGCCGACGGGAGCGAGGAGATGGAAGCAGTCGCTATCATCGACGTTTTGAAACGCGCAA
AAGCTAACGTTGTCGTGGCCGCGTTGGGCAATTCTCTTGAGGTCGTTGCATCTCGTAAAGTGAAA
CTTGTCGCTGACGTATTATTAGATGAAGCGGAGAAAAACAGCTATGATTTAATCGTTTTGCCGGG
AGGTCTGGGCGGGGCTGAAGCATTCGCATCCTCAGAAAAATTGGTTAATATGCTTAAGAAACAAG
CAGAATCTAATAAGCCTTATGGTGCTATTTGTGCTTCCCCCGCTCTTGTGTTTGAGCCTCATGGA
CTTCTTAAGGGCAAAAAGGCTACTGCCTTCCCAGCTATGTGTTCGAAATTAACAGATCAGTCTCA
CATCGAACATCGTGTCTTGGTTGACGGGAACCTTATTACCTCCCGTGGACCGGGTACTTCATTGG
AATTTGCTTTGGCTATCGTCGAAAAATTTTACGGGCGTGAAAAGGGGTTGCAGTTATCGAAGGCA
ACTCTGGTG
```

AtDJ-1B protein sequence (after cleavage of GST fusion tag), catalytic cysteines are underlined

(UniProt Accession Q9MAH3)

```
GATMSSSTKK VLIPVAHGTE PFEAVVMIDV LRRGGADVTV ASVENQVGVD
ACHGIKMVAD TLLSDITDSV FDLIMLPGGL PGGETLKNCK PLEKMVKKQD
TDGRLNAAIC CAPALAFGTW GLLEGKKATC YPVFMEKLAA CATAVESRVE
IDGKIVTSRG PGTTMEFSVT LVEQLLGKEK AVEVSGPLVM RPNPGDEYTI
TELNQVSWSF EGTPQILVPI ADGSEEMEAV AIIDVLKRAK ANVVVAALGN
SLEVVASRKV KLVADVLLDE AEKNSYDLIV LPGGLGGAEA FASSEKLVNM
LKKQAESNKP YGAICASPAL VFEPHGLLKG KKATAFPAMC SKLTDQSHIE
HRVLVDGNLI TSRGPGTSLE FALAIVEKFY GREKGLQLSK ATLV
```

Table S4. Primers used for the study.

ID	Sequence	Use
	DJ-1 overexpression in E. coli	
**DJ1B-attB1-TEV-Fw**	GGGGACAAGTTTGTACAAAAAAGCAGGC TTCATGGAAAACCTGTATTTTCAGGGAGC GACGATGTCGAGCAGTACGAAAAAGGTA CTTATT	PCR for gene amplification and adding TEV site
**DJ1B-attB2-Rev**	GGGGACCACTTTGTACAAGAAAGCTGGG TCTTACACCAGAGTTGCCTTCGATA	PCR for gene amplification and adding TEV site
**SeqLA**	CTCTCGCGTTAACGCTAGCATGGAT	Sequencing of the construct in pDONR221
**SeqLB**	GTAACATCAGAGATTTTGAGACAC	Sequencing of the construct in pDONR221
**SeqFw2**	GGTGGAAGTTTCAGGGCCCCTGGT	Sequencing of the construct in pDONR221

**SeqRev2**	ACCAGGGGCCCTGAAACTTCCACC	Sequencing of the construct in pDONR221
	Analysis of T-DNA insertion lines	
**SALK_049637_DJ1A_F**	CCTCCCTTTTCCCAATCATATC	Genotyping the *dj1a* (SALK_049637) T-DNA line
**SALK_049637_DJ1A_R**	TTTTTCGACCGGTTAACACTC	Genotyping the *dj1a* (SALK_049637) T-DNA line
**SALK_093414_DJ1B_F**	AGGCACAAATTGCTCCATATG	Genotyping the *dj1b-9* (SALK_093414) T-DNA line
**SALK_093414_DJ1B_R**	ACCATGGAATTCTCTGTCACG	Genotyping the *dj1b-9* (SALK_093414) T-DNA line
**SALK_046449_DJ1B_F**	GACGCATGAGCTCAGTAAAGC	Genotyping the *dj1b-4* (SALK_046449) T-DNA line
**SALK_046449_DJ1B_R**	AGCAAGACACTGATGGACGAC	Genotyping the *dj1b-4* (SALK_046449) T-DNA line
**LB_SALK**	ATTTTGCCGATTTCGGAAC	Genotyping the *dj1b-4* and *dj1b-9* SALK T-DNA lines
**DJ1A_qPCR_F1**	GGCGGGCAAAAGCAAATGTA	rt-qPCR analysis of DJ-1A expression
**DJ1A_qPCR_R1**	AAGACCGCCAGGTAACACAA	rt-qPCR analysis of DJ-1A expression
**DJ1A_qPCR_F2**	TGATTGTGTTACCTGGCGGT	rt-qPCR analysis of DJ-1A expression
**DJ1A_qPCR_R2**	AGGCTCGAAGACGTAAGCAG	rt-qPCR analysis of DJ-1A expression
**DJ1B_qPCR_F1**	GAAGCAGGCGGAATCAAACA	rt-qPCR analysis of DJ-1B expression
**DJ1B_qPCR_R1**	GTTGCCTTCTTACCCTTGAGT	rt-qPCR analysis of DJ-1B expression
**DJ1B_qPCR_F2**	GGTTTACTCAAGGGTAAGAAGGC	rt-qPCR analysis of DJ-1B expression
**DJ1B_qPCR_R2**	GAGATTGCCGTCCACCAAGA	rt-qPCR analysis of DJ-1B expression
**DJ1B_qPCR_F3**	CTCATGGTACGGAGCCGTTT	rt-qPCR analysis of DJ-1B expression
**DJ1B_qPCR_R3**	GGAAGTCCTCCAGGGAGCATA	rt-qPCR analysis of DJ-1B expression

Table S5. Log2 fold change values of DJ-1 mRNA expression levels, as visualised on Figure 9.

Gene	AtDJ-1A	AtDJ-1B	AtDJ-1C	AtDJ-1D	AtDJ-1E	AtDJ-1F
	**At3g14990**	**At1g53280**	**At4g34020**	**At3g02720**	**At2g38860**	**At3g54600**
***Pseudomonas syringae* infection (Stael et al., personal communication)**	1.376	−0.901	−2.673	−1.516	2.601	−4.146
**3h high light, *cat2-2* [27]**	2.859	−0.357	−1.201	−0.456	1.86	−2.137
**Methyl viologen, 24 h (He et al., submitted)**	0.873	0.117	−0.04	0.046	0.263	0.363
***cat2-2* vs. Col-0 [30]**	1.688	0.105	−0.033	0.155	1.214	0.277
***cat2-2* 24h RGCL [30]**	2.945	−0.204	−0.112	−0.56	1.877	−3.988
**Col-0 25h RGCL [30]**	3.115	0.615	0.473	−0.209	0.888	−2.354
**50 µM Antimycin A [28]**	0.958	−0.155	−0.275	−0.353	0.282	−1.357

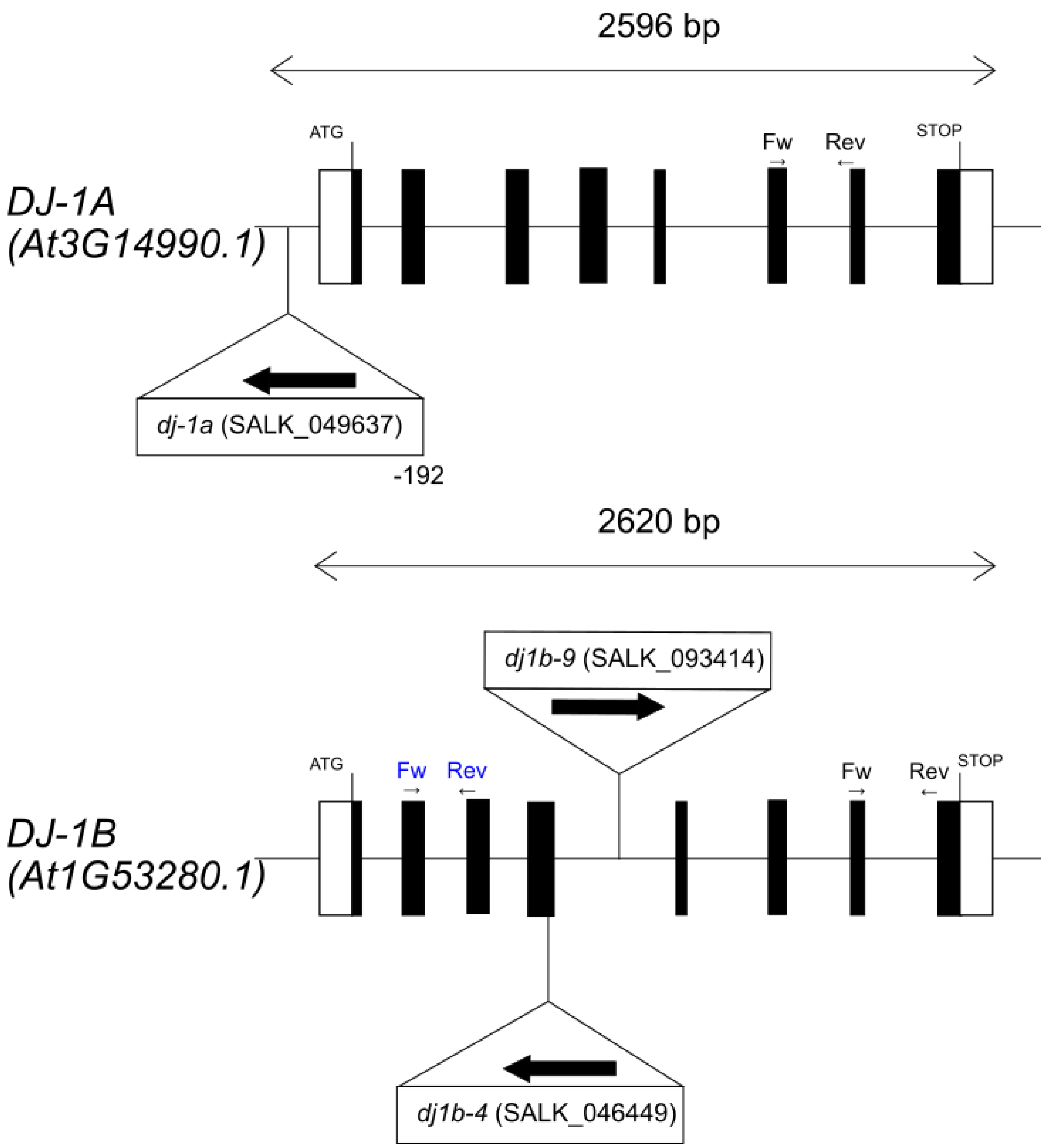

Figure S1. *AtDJ-1A* and *AtDJ-1B* gene models. For *AtDJ-1A* and *AtDJ-1B* two pairs of primers complementary to C-terminal fragment of the transcript were used (plasmids with suffix: qPCR_F1/R1/F2/R2), their positions marked in black (Fw, Rev). For *AtDJ-1B* an additional primer pair complementary to the N-terminal fragment was used: qPCR_F3/R3, marked in blue.

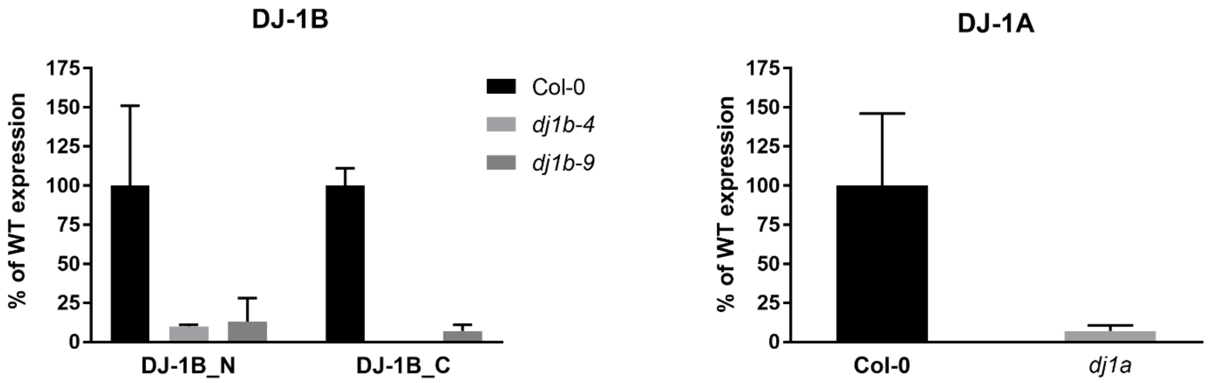

Figure. S2. *AtDJ-1B* and *DJ-1A* transcript levels (left and right, respectively) in WT and KO T-DNA lines. DJ1B_N represents the transcript detected with primers complementary to N-terminal fragment of DJ-1B mRNA (blue arrows, Figure S1), DJ1B_C represents the transcript detected with primers complementary to C-terminal fragment of *AtDJ-1B* mRNA (black arrows, Figure S1). RNA was extracted from pooled 11-day-old plants grown *in vitro* and used to quantify gene expression levels by RT-qPCR. Values are means ± SD.
